# Postdialysis blood pressure rise predicts long-term outcomes in chronic hemodialysis patients: a four-year prospective observational cohort study

**DOI:** 10.1186/1471-2369-13-12

**Published:** 2012-03-14

**Authors:** Chih-Yu Yang, Wu-Chang Yang, Yao-Ping Lin

**Affiliations:** 1 Division of Nephrology, Department of Medicine, Taipei Veterans General Hospital, No. 201, Section 2, Shih-Pai Road, Taipei 11217, Taiwan; 2 School of Medicine, National Yang-Ming University, Taipei, Taiwan

**Keywords:** Postdialysis blood pressure, Systolic blood pressure, Cardiothoracic ratio, Hemodialysis, Mortality

## Abstract

**Background:**

The blood pressure (BP) of a proportion of chronic hemodialysis (HD) patients rises after HD. We investigated the influence of postdialysis BP rise on long-term outcomes.

**Methods:**

A total of 115 prevalent HD patients were enrolled. Because of the fluctuating nature of predialysis and postdialysis BP, systolic BP (SBP) and diastolic BP before and after HD were recorded from 25 consecutive HD sessions during a 2-month period. Patients were followed for 4 years or until death or withdrawal.

**Results:**

Kaplan-Meier estimates revealed that patients with average postdialysis SBP rise of more than 5 mmHg were at the highest risk of both cardiovascular and all-cause mortality as compared to those with an average postdialysis SBP change between -5 to 5 mmHg and those with an average postdialysis SBP drop of more than 5 mmHg. Furthermore, multivariate Cox regression analysis indicated that both postdialysis SBP rise of more than 5 mmHg (HR, 3.925 [95% CI, 1.410-10.846], *p *= 0.008) and high cardiothoracic (CT) ratio of more than 50% (HR, 7.560 [95% CI, 2.048-27.912], *p *= 0.002) independently predicted all-cause mortality. We also found that patients with an average postdialysis SBP rise were associated with subclinical volume overload, as evidenced by the significantly higher CT ratio (*p *= 0.008).

**Conclusions:**

A postdialysis SBP rise in HD patients independently predicted 4-year cardiovascular and all-cause mortality. Considering postdialysis SBP rise was associated with higher CT ratio, intensive evaluation of cardiac and volume status should be performed in patients with postdialysis SBP rise.

## Background

Due to oliguria or even anuria, most end-stage renal disease (ESRD) patients undergoing maintenance hemodialysis (HD) require ultrafiltration during HD in order to maintain a euvolemic status. Although the volume-dependent component of hypertension may be corrected by fluid removal, a proportion of HD patients experience postdialysis BP rise. The underlying mechanisms of intradialytic hypertension are complex and have been considered to be caused by clinically silent fluid overload, activation of the renin-angiotensin axis, sympathetic overactivity, endothelial dysfunction, and sodium loading during HD [1-8]. Inrig *et al*. recently recognized its association with endothelial cell dysfunction, which was assessed by peripheral blood endothelial progenitor cells and ultrasonographic measurement of brachial artery flow-mediated vasodilation underlie the pathogenesis of intradialytic hypertension [9].

Cardiovascular disease is the leading cause of morbidity and mortality in ESRD [10]. Hypertension is highly prevalent in patients undergoing HD and contributes to the high cardiovascular morbidity and mortality in these patients [11]. Postdialysis BP goals should be below 130/80 mmHg according to the National Kidney Foundation's Kidney Disease Outcomes Quality Initiative (KDOQI) recommendation [12]. Surprisingly, little attention had been paid on postdialysis hypertension until some recent studies showed its clinical significance using BP recordings from 3-6 HD sessions [1,4,13]. However, the predictive values of predialysis and postdialysis BP have been disputed due to its high variability nature [14-16], which could be overcome by increasing the number of BP recording times for more than 1 month [17,18].

Although some studies have demonstrated the adverse impact of intradialytic hypertension on 2-year all-cause mortality [1,4], the relationship between postdialysis hypertension and cardiovascular outcomes was unknown. We hypothesized that poor overall survival in such patients is related to volume overload that leading to future cardiovascular death. We also aimed to elucidate whether the adverse outcomes persist with higher follow-up. Therefore, we followed up our HD patients for more than 4 years and included cardiovascular mortality in patient outcomes as well.

## Methods

### Study protocol and subjects

From January 2006 to June 2010, we conducted a prospective observational cohort study at the Taipei Veterans General Hospital, a tertiary-care referral hospital. Each enrolled subject provided written informed consent, and this study was approved by the Institutional Review Board of Taipei Veterans General Hospital. All patients were at least 18 years of age, had ESRD, and had been on maintenance HD for at least 3 months. At study entry in 2006, we screened all prevalent HD patients in our unit (*n *= 178). We excluded recent adverse cardiovascular events or hospitalization within 3 months (*n *= 7), signs of current infection (*n *= 7), active autoimmune disease/collagen vascular disease (*n *= 1), advanced/severe liver disease (*n *= 2), patients received HD only twice weekly (*n *= 12), and patients who refused to provide consent (*n *= 34). Eventually, a total of 115 prevalent HD patients were enrolled.

At study entry, we recorded predialysis and postdialysis systolic BP (SBP) and diastolic BP (DBP) for 25 consecutive HD sessions during a 2-month period. The demographic features and clinical parameters, including age, gender, duration of dialysis, body weight index, comorbidities, nutritional supplements, medications, serum biochemical data, and blood cell counts were obtained. Smoking status was defined as never (0), quit (1), or current smoker (2). Cardiothoracic (CT) ratios were obtained from postdialysis chest radiographs in a standing position (posterior-anterior view). The CT ratio was calculated by dividing the maximal transverse diameter of the cardiac silhoutte by the transverse inner diameter of the rib cage. It was interpreted by an independent radiologist and two independent nephrologists separately, and was then averaged from three data of each patient to reflect the volume status.

### Dialysis procedures

HD was performed three times weekly (4 hours per session) using 1.8-m_2 _surface area dialyzers with bicarbonate-based dialysates (sodium, 140 mEq/L [mmol/L]; bicarbonate, 39 mEq/L [mmol/L]; potassium, 2.0 mEq/L [mmol/L]; calcium, 3.0 mEq/L; and magnesium 1.0 mEq/L). All patients were treated with recombinant human erythropoietin at an average dosage of 20,000 units monthly, with a target hematocrit level of 30-36%.

### BP measurements

Brachial artery BP was measured with the mercury sphygmomanometer in seated position. Predialysis BP was measured at the beginning of HD after 15 minutes of quiet rest. Postdialysis BP was measured at the end of HD 15 minutes after disconnecting from the dialysis circuit. Both predialysis and postdialysis BP were measured in the seated position. Mean arterial pressure (MAP) was calculated as (SBP + 2 × DBP)/3. Pulse pressure (PP) was calculated from the SBP and DBP (PP = SBP - DBP). Postdialysis BP rise or drop was defined by the difference between postdialysis and predialysis BP values. The variability of postdialysis SBP alteration of each patient was also examined. We first calculated the percentage of postdialysis SBP alteration in each HD sessions as (postdialysis SBP - predialysis SBP)/predialysis SBP * 100%. A total of 25 postdialysis SBP alteration percentages were obtained from each patient. We further calculated the standard deviation of these 25 data, which was defined as the variability of postdialysis SBP alteration of each patient. Ultrafiltration volume was defined as the amount of fluid removed within an HD session. All of the BP values and ultrafiltration volume were averaged from 25 consecutive HD sessions during a 2-month period.

### Laboratory measurements

Blood samples were collected before the mid-week HD session. Samples were centrifuged within 1 hour of collection and then immediately sent to the central laboratory for analysis. Laboratory data were recorded as the average of 3 months' data. All measurements were determined in a single central laboratory.

### Clinical outcomes

After baseline assessments, all patients were followed for 4 years or until death. Patients who received a kidney transplantation were censored at the time of transplantation. Clinical outcomes were death due to non-cardiovascular etiologies and cardiovascular diseases, including acute myocardial infarction, sudden cardiac arrest, fatal arrhythmia, cardiogenic shock, and stroke. For cardiovascular mortality, patients were also censored at the time of other causes of death. The causes of death were determined by the attending physicians who had no knowledge of patient grouping according to postdialysis BP alterations. In cases of death that did not occur in our hospital, family members were interviewed by telephone to ascertain the cause and time of death.

### Statistical analysis

Chi-square analysis or Fisher's exact test was used for comparison of categorical variables as appropriate. Continuous variables were compared by analysis of variance (ANOVA), Student's *t*-test, or paired *t*-test as appropriate. Values of the continuous variables are presented as mean and standard deviation, unless otherwise specified. The Cox proportional hazards model was used to determine the significance of variables in predicting the primary end-point, including all-cause mortality and cardiovascular mortality. Variables associated with clinical outcomes in univariate Cox regression analysis with *p *values less than 0.10 were used for multivariate Cox regression analysis. Kaplan-Meier analysis was used to assess the difference among patients with a postdialysis BP rise of more than 5 mmHg, those with a postdialysis SBP change between -5 to 5 mmHg, and those with a postdialysis BP drop of more than 5 mmHg in reaching the primary end-point. This comparison was performed using the log-rank test. SPSS version 15.0 for Windows (SPSS Inc., Chicago, Illinois, USA) was used for all statistical analyses. All probabilities were two-tailed and a *p *value of less than 0.05 was considered statistically significant.

## Results

### Baseline characteristics of study subjects

Table 1 shows the baseline characteristics of the 115 patients who were followed for 4 years. At enrollment, the mean age was 64 years, 45% of patients were male, and the mean duration on dialysis was 5.8 years. A total of 46 patients (40%) had diabetes mellitus, 105 patients (91%) had hypertension, 30 patients (26%) had cardiovascular diseases, 5 patients (4%) had a history of stroke, and 9 patients (8%) had malignancy. BP before and after HD and ultrafiltration volume were averaged from 25 consecutive HD sessions during a 2-month period. We categorized our patients into 3 groups, including patients with an average postdialysis SBP rise of more than 5 mmHg (*n *= 39), those with an average postdialysis SBP change between -5 to 5 mmHg (*n *= 27), and those with an average postdialysis SBP drop of more than 5 mmHg (*n *= 49).

**Table 1:** Demographic characteristics of the study population

Factor	All	Patients with postdialytic SBP rise > 5 mmHg	Patients with postdialytic SBP change between -5 to 5 mmHg	Patients with postdialytic SBP drop > 5 mmHg	*p *Value
Patient number	115	39	27	49	
Age (year)	64.0 ± 13.6	68.6 ± 11.5	65.1 ± 16.0	59.6 ± 12.6	0.007*
Male gender (%)	45.2	41.0	33.3	55.1	0.153
Dialysis duration (year)	5.8 ± 5.3	4.5 ± 5.3	6.8 ± 6.1	6.2 ± 4.7	0.180
Body mass index (Kg/m^2^)	22.0 ± 3.5	21.7 ± 3.4	21.9 ± 3.6	22.2 ± 3.5	0.840
Smoking index	0.46 ± 0.74	0.46 ± 0.79	0.41 ± 0.69	0.49 ± 0.74	0.900
Kt/V	1.60 ± 0.26	1.61 ± 0.22	1.62 ± 0.22	1.59 ± 0.31	0.873
Comorbidities (%)					
Diabetes mellitus (%)	40.0	43.6	37.0	38.8	0.844
Hypertension (%)	91.3	89.7	92.6	91.8	1.000
Cardiovascular disease (%)	26.1	30.8	29.6	20.4	0.487
Prior stroke (%)	4.3	7.7	0.0	4.1	0.365
Malignancy (%)	7.8	15.4	3.7	4.1	0.136
Medications					
ACE inhibitor/ARB (%)	47.0	53.8	44.4	42.9	0.565
Antihypertensive agents (%)	79.1	89.7	85.2	67.3	0.025*
Antiplatelet agents (%)	33.0	41.0	33.3	26.5	0.356
Statins (%)	35.7	33.3	37.0	36.7	0.933
Fibrates (%)	7.0	10.3	3.7	6.1	0.719
Vitamin D_3 _(%)	19.1	15.4	18.5	22.4	0.702
Hepatitis B infection (%)	7.8	7.7	0.0	12.2	0.172
Hepatitis C infection (%)	18.3	12.8	25.9	18.4	0.409
Laboratory data					
Albumin (g/dL)	4.1 ± 0.3	4.1 ± 0.3	4.2 ± 0.3	4.2 ± 0.3	0.224
Total cholesterol (mg/dL)	173.5 ± 36.1	175.4 ± 35.0	171.1 ± 31.4	173.4 ± 39.9	0.895
Triglycerides (mg/dL)	182.9 ± 125.2	166.6 ± 113.1	190.8 ± 145.1	191.4 ± 123.9	0.614
Sodium (mmol/L)	139.8 ± 2.9	139.4 ± 2.9	139.9 ± 3.5	140.0 ± 3.1	0.693
Chloride (mmol/L)	99.7 ± 2.5	100.1 ± 2.3	100.0 ± 2.6	99.1 ± 2.6	0.148
Bicarbonate (mmol/L)	24.0 ± 2.5	24.1 ± 2.5	23.7 ± 2.3	24.1 ± 2.6	0.770
ALT (U/L)	20.4 ± 12.5	20.5 ± 12.1	23.0 ± 18.4	18.8 ± 8.0	0.460
Alk-P (mg/dL)	113.9 ± 61.5	117.3 ± 79.3	103.0 ± 42.4	117.1 ± 54.2	0.578
Fasting glucose (mg/dL)	139.7 ± 66.6	143.3 ± 57.0	140.2 ± 75.8	136.4 ± 69.6	0.890
HbA_1c _(%)	6.2 ± 1.5	6.0 ± 1.1	6.0 ± 1.6	6.3 ± 1.8	0.573
WBC count (1000 per cumm)	6.3 ± 1.8	6.2 ± 1.6	6.4 ± 1.6	6.5 ± 2.0	0.741
Hemoglobin (g/dL)	10.1 ± 1.3	9.7 ± 1.0	10.2 ± 1.6	10.3 ± 1.4	0.077
High-sensitive CRP (mg/dL)	0.514 ± 0.706	0.573 ± 0.662	0.343 ± 0.305	0.562 ± 0.875	0.357
Ferritin (ng/mL)	510.3 ± 570.7	508.5 ± 541.1	595.1 ± 944.2	464.9 ± 229.4	0.723
Transferrin saturation (%)	26.3 ± 11.7	24.8 ± 10.8	29.9 ± 15.7	25.4 ± 9.5	0.178
Peridialytic blood pressure					
predialytic SBP (mmHg)	143.1 ± 15.5	138.1 ± 16.6	141.5 ± 13.8	147.9 ± 14.3	0.009*
postdialytic SBP (mmHg)	143.1 ± 17.1	152.6 ± 19.2	142.7 ± 14.5	135.7 ± 12.6	< 0.001*
predialytic DBP (mmHg)	78.2 ± 6.8	74.9 ± 6.9	77.9 ± 5.8	81..0 ± 6.1	< 0.001*
postdialytic DBP (mmHg)	79.1 ± 6.2	80.0 ± 6.8	79.6 ± 6.6	78.2 ± 5.4	0.345
predialytic MAP (mmHg)	99.8 ± 9.1	96.0 ± 9.6	99.1 ± 7.7	103.3 ± 8.1	0.001*
postdialytic MAP (mmHg)	100.5 ± 9.0	104.2 ± 10.1	100.6 ± 8.4	97.3 ± 7.2	0.001*
predialytic PP (mmHg)	64.9 ± 11.4	63.2 ± 11.8	63.6 ± 11.1	67.0 ± 11.1	0.238
Postdialytic PP (mmHg)	64.0 ± 13.8	72.6 ± 15.2	63.2 ± 11.4	57.5 ± 9.8	< 0.001*
Ultrafiltration volume (Kg)	2.4 ± 0.8	2.1 ± 0.5	2.3 ± 0.9	2.6 ± 0.8	0.006*
CT ratio > 50% (%)	68.7	89.7	63.0	55.1	0.002*

Values are expressed as mean ± SD or percent unless otherwise listed. Abbreviations: ACE, angiotensin converting enzyme; ARB, angiotensin II-receptor blocker; ALT, alanine aminotransferase; Alk-P, alkaline phosphatase; HbA1c, glycosylated hemoglobin; WBC, white blood cell; CRP, C-reactive protein; SBP, systolic blood pressure; DBP, diastolic blood pressure; MAP, mean arterial pressure; PP, pulse pressure; CT, cardiothoracic. *: *p *< 0.05.

Compared to patients with an average postdialysis SBP change between -5 to 5 mmHg and those with an average postdialysis SBP drop for more than 5 mmHg, those with an average postdialysis SBP rise of more than 5 mmHg were more likely to be older (*p *= 0.007), have a higher percentage of antihypertensive agents usage (*p *= 0.025), lower hemoglobin level (*p *= 0.077), smaller ultrafiltration volume (*p *= 0.006), and higher CT ratio (*p *= 0.002).

**Figure 1:**
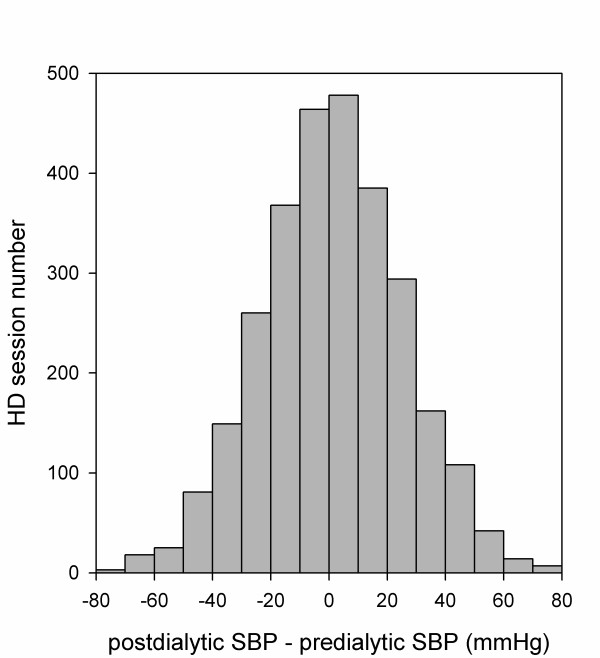
**Histogram of postdialysis SBP change of 2875 HD sessions in 115 patients**. Abbreviations: SBP, systolic blood pressure; HD, hemodialysis.

**Figure 2:**
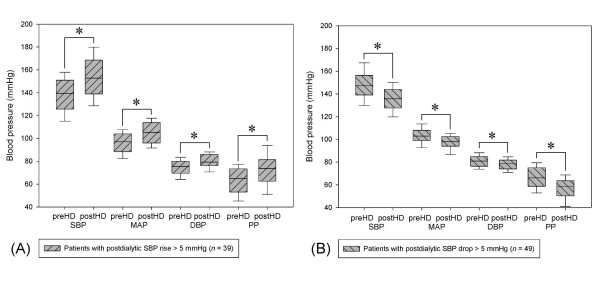
**Predialysis and postdialysis blood pressure categorized by postdialysis SBP rise > 5 mmHg (*n *= 39) (A) and those with postdialysis SBP drop > 5 mmHg (*n *= 49) (B)**. Abbreviations: HD, hemodialysis; SBP, systolic blood pressure; MAP, mean arterial pressure; DBP, diastolic blood pressure; PP, pulse pressure. (*: *p *< 0.001).

Figure 1 shows the histogram of postdialysis SBP alterations of 2875 HD sessions in 115 patients. Among all of the patients, the average SBP of 39 patients (33.9%) rose more than 5 mmHg after dialysis. In addition, the average MAP of 31 patients (27.0%), DBP of 23 patients (20.0%), and PP of 32 patients (27.8%) rose more than 5 mmHg after dialysis. Figure 2 shows the differences between predialysis and postdialysis BP in patients with an average postdialysis SBP rise of more than 5 mmHg (*n *= 39) and those with an average postdialysis SBP drop of more than 5 mmHg (*n *= 49). For patients with an average postdialysis BP for more than 5 mmHg, the average predialysis BPs were 138 ± 17 mmHg (range, 103 to 164 mmHg) for SBP, 96 ± 10 mmHg (range, 76 to 112 mmHg) for MAP, 75 ± 7 mmHg (range, 61 to 87 mmHg) for DBP, and 63 ± 12 mmHg (range, 40 to 85 mmHg) for PP. The average postdialysis BPs were 153 ± 19 mmHg (range, 109 to 182 mmHg) for SBP, 104 ± 10 mmHg (range, 81 to 119 mmHg) for MAP, 80 ± 7 mmHg (range, 67 to 96 mmHg) for DBP, and 73 ± 15 mmHg (range, 42 to 102 mmHg) for PP.

### Relationship between postdialysis BP rise and long-term clinical outcomes

The mean follow-up time was 3.4 ± 1.4 years. By the censoring date, 40 patients (35%) had died, of whom 18 (16%) were related to cardiovascular etiologies. The causes of cardiovascular death were acute myocardial infarction (7 patients), sudden cardiac arrest suspected fatal arrhythmia (6 patients), critical aortic stenosis (3 patients), and stroke (2 patients). Among patients who died due to non-cardiovascular mortality, the causes of mortality were sepsis (16 patients), malignancy (4 patients), severe gastrointestinal bleeding (1 patient), and hollow organ perforation (1 patient). Seven patients were given kidney transplantations. Univariate Cox regression analysis indicated that patients with an average postdialysis SBP rise of more than 5 mmHg were more likely to suffer from cardiovascular mortality (HR, 3.756 [95% CI, 1.454-9.703], *p *= 0.006) and all-cause mortality (HR, 2.382 [95% CI, 1.280-4.434], *p *= 0.006) than those without. However, the variability of postdialysis SBP alteration was not associated with all-cause mortality (HR, 0.949 [95% CI, 0.872-1.033], *p *= 0.230) or cardiovascular mortality (HR, 1.004 [95% CI, 0.908-1.109], *p *= 0.945).

### Postdialysis SBP rise was associated with both cardiovascular and all-cause mortality

**Figure 3:**
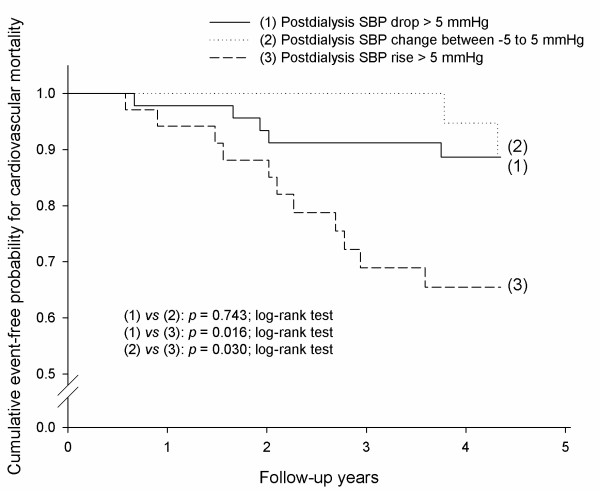
**Hemodialysis patients with an average postdialysis systolic blood pressure (SBP) rise of more than 5 mmHg were at the highest risk of cardiovascular mortality, as compared to those with an average postdialysis SBP change between -5 to 5 mmHg (*p *= 0.030; log-rank test) and those with an average postdialysis SBP drop > 5 mmHg (*p *= 0.016; log-rank test)**.

**Figure 4:**
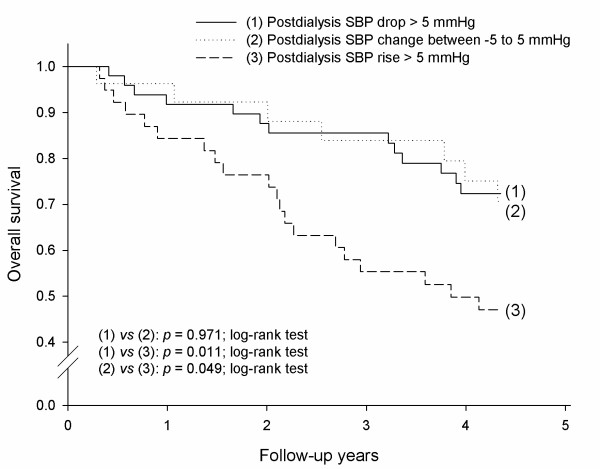
**Hemodialysis patients with an average postdialysis systolic blood pressure (SBP) rise of more than 5 mmHg were at the highest risk for all-cause mortality, as compared to those with an average postdialysis SBP change between -5 to 5 mmHg (*p *= 0.049; log-rank test) and those with an average postdialysis SBP drop > 5 mmHg (*p *= 0.011; log-rank test)**.

Kaplan-Meier estimates showed a significantly greater cardiovascular mortality rate for patients with an average postdialysis SBP rise of more than 5 mmHg than for those with an average postdialysis SBP change between -5 to 5 mmHg (*p *= 0.030; log-rank test) and for those with an average postdialysis SBP drop of more than 5 mmHg (*p *= 0.016; log-rank test) (Figure 3). In addition, patients with an average postdialysis SBP rise of more than 5 mmHg had a significantly higher all-cause mortality rate than those with an average postdialysis SBP change between -5 to 5 mmHg (*p *= 0.049; log-rank test) and those with an average postdialysis SBP drop of more than 5 mmHg (*p *= 0.011; log-rank test) (Figure 4).

### Significant predictors of all-cause mortality by multivariate Cox regression analysis

As shown in Table 2, initial univariate Cox regression analysis of variables significantly associated with all-cause mortality (*p *< 0.10) indicated that old age, male gender, low Kt/V, diabetes mellitus, cardiovascular disease, underlying malignancy, hypoalbuminemia, high fasting blood glucose, elevated high-sensitive C-reactive protein (CRP), average postdialysis SBP rise of more than 5 mmHg, large ultrafiltration volume, and high CT ratio increased the risk. We included all these variables in a multivariate Cox regression analysis. The result indicated that old age, male gender, hypoalbuminemia, elevated baseline high-sensitive CRP, large ultrafiltration volume, high CT ratio, and average postdialysis SBP rise of more than 5 mmHg were independent predictors of 4-year all-cause mortality.

**Table 2:** Univariate and multivariate Cox regression analysis of significant predictors of 4-year all-cause mortality in hemodialysis patients

Factor	Univariate				Multivariate			
	
	HR	95% CI		*p *Value	HR	95% CI		*p *Value
						
		Lower	Upper			Lower	Upper	
Age (10 year)	1.713	1.284	2.286	< 0.001*	1.947	1.366	2.775	< 0.001
Male gender (%)	1.899	1.014	3.557	0.045*	4.267	1.985	9.171	< 0.001
Dialysis duration (year)	0.951	0.889	1.017	0.140				
Body mass index (Kg/m^2^)	1.000	0.914	1.093	0.995				
Smoking index	1.167	0.795	1.712	0.430				
Kt/V	0.116	0.033	0.416	0.001*				
Comorbidities (%)								
Diabetes mellitus (%)	2.125	1.138	3.967	0.018*				
Hypertension (%)	1.352	0.417	4.388	0.615				
Cardiovascular disease (%)	2.256	1.204	4.227	0.011*				
Prior stroke (%)	2.588	0.797	8.406	0.114				
Malignancy (%)	2.704	1.130	6.467	0.025*				
Hepatitis B infection (%)	0.887	0.273	2.879	0.842				
Hepatitis C infection (%)	0.896	0.376	2.135	0.805				
Medications								
Antihypertensive agents (%)	0.921	0.439	1.935	0.829				
ACE inhibitor/ARB (%)	1.141	0.614	2.121	0.676				
Vitamin D_3 _(%)	1.156	0.533	2.510	0.713				
Laboratory data								
Albumin (g/dL)	0.136	0.044	0.425	0.001*	0.032	0.008	0.126	< 0.001
Total cholesterol (mg/dL)	0.997	0.989	1.006	0.509				
Triglycerides (mg/dL)	1.001	0.999	1.003	0.442				
Sodium (mmol/L)	0.937	0.841	1.044	0.236				
Chloride (mmol/L)	0.984	0.869	1.114	0.801				
Bicarbonate (mmol/L)	0.959	0.851	1.082	0.497				
ALT (U/L)	1.001	0.978	1.024	0.963				
Alk-P (mg/dL)	1.000	0.995	1.005	0.927				
Fasting glucose (mg/dL)	1.004	1.000	1.008	0.030*				
HbA_1c _(%)	1.135	0.956	1.347	0.149				
WBC count (1000 per cumm)	0.958	0.788	1.166	0.671				
Hemoglobin (g/dL)	1.019	0.806	1.289	0.873				
High-sensitive CRP (mg/dL)	1.432	1.062	1.932	0.019*	1.534	1.072	2.195	0.019
Ferritin (ng/mL)	1.000	1.000	1.001	0.743				
Transferrin saturation (%)	0.826	0.065	10.479	0.883				
Peridialytic BP								
predialytic SBP (mmHg)	1.000	0.980	1.020	0.993				
postdialytic SBP (mmHg)	1.008	0.990	1.026	0.393				
predialytic MAP (mmHg)	0.992	0.959	1.027	0.648				
postdialytic MAP (mmHg)	1.003	0.970	1.038	0.844				
predialytic DBP (mmHg)	0.978	0.934	1.024	0.346				
postdialytic DBP (mmHg)	0.981	0.932	1.032	0.452				
predialytic PP (mmHg)	1.008	0.981	1.035	0.578				
postdialytic PP (mmHg)	1.016	0.994	1.038	0.159				
Peridialytic BP alterations (no as reference)								
postdialytic SBP rise > 5 mmHg (yes/no)	2.382	1.280	4.434	0.006*	3.925	1.420	10.846	0.008
postdialytic MAP rise > 5 mmHg (yes/no)	1.762	0.929	3.344	0.083				
postdialytic DBP rise > 5 mmHg (yes/no)	1.602	0.800	3.207	0.184				
postdialytic PP rise > 5 mmHg (yes/no)	1.548	0.808	2.965	0.188				
Ultrafiltration volume (Kg)	1.355	0.944	1.944	0.099*	3.470	2.045	5.889	< 0.001
CT ratio > 50% (yes/no)	8.960	2.322	34.572	0.001*	7.560	2.048	27.912	0.002

Abbreviations: HR, Hazard ratio; CI, confidence interval; ACE, angiotensin converting enzyme; ARB, angiotensin II-receptor blocker; ALT, alanine aminotransferase; Alk-P, alkaline phosphatase; HbA1c, glycosylated hemoglobin; WBC, white blood cell; CRP, C-reactive protein; BP, blood pressure; SBP, systolic blood pressure; MAP, mean arterial pressure; DBP, diastolic blood pressure; PP, pulse pressure; CT, cardiothoracic. *Variables further included in the multivariate Cox regression analysis.

### Positive correlation between postdialysis SBP rise and high CT ratio

**Figure 5:**
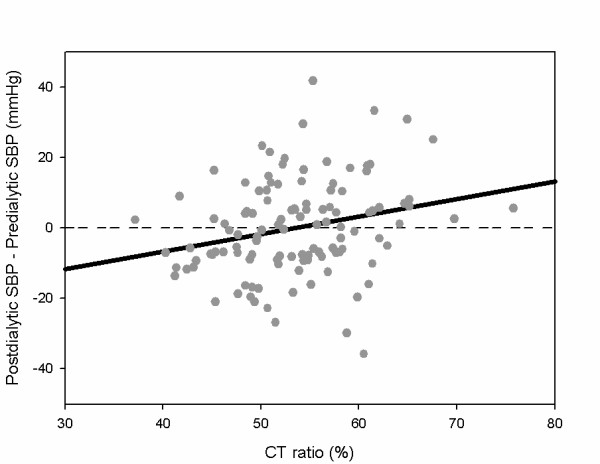
**Scatter plot showing the positive correlation between postdialysis SBP alterations and CT ratio (r = 0.247, *p *= 0.008)**. Abbreviations: SBP, systolic blood pressure; CT, cardiothoracic.

As shown in Table 1, among patients with an average postdialysis SBP rise of more than 5 mmHg, up to 89.7% suffered from a high CT ratio (more than 50%). This showed that patients with an average postdialysis SBP rise might be associated with subclinical volume overload, and/or might suffer from cardiomyopathy. We found a significant positive correlation between postdialysis SBP rise and high CT ratio (r = 0.247, *p *= 0.008) (Figure 5). However, there were still some patients with a decline in SBP during HD had a high CT ratio. This is consistent with the above findings of multivariate analysis that both postdialysis SBP rise and high CT ratio are independent outcome predictors.

## Discussion

In chronic HD patients, there are three different time periods for BP recordings: peridialysis, intradialysis, and interdialysis [19]. The optimal BP of chronic HD patients recommended by the KDOQI guidelines is based on peridialysis BP, rather than intradialytic or interdialysis BP [12]. Previous studies have shown a reverse epidemiology association between absolute predialysis and postdialysis BP values and clinical outcomes [18,20]. In the present study, we examined the relationship between peridialysis BP alterations and outcomes, which revealed that patients with an average postdialysis SBP rise of more than 5 mmHg were more likely to suffer from 4-year cardiovascular and all-cause mortality. A higher CT ratio was also an independent long-term outcome predictor in our study. This is consistent with previous studies which showed that a CT ratio of more than 50% was associated with 2-year mortality in chronic HD patients [21,22]. Meanwhile, we found that among patients with an average postdialysis SBP rise of more than 5 mmHg, up to 89.7% suffered from a high CT ratio. This suggests that the prevalence of subclinical fluid overload is high in chronic HD patients, and that volume excess may manifest as postdialysis hypertension. Furthermore, previous outcome studies showing that intradialytic hypertension was associated with 2-year all-cause mortality did not examined cardiovascular outcomes [1,4]. To the best of our knowledge, our study is the first to demonstrate the positive correlation between postdialysis SBP rises and long-term cardiovascular mortality.

Our data also showed that among patients with postdialysis SBP rise, their MAP, DBP, and PP also rose (Figure 2). However, after multivariate Cox regression analysis, only SBP remained significant in predicting outcomes. Patients with an average postdialysis SBP rise of more than 5 mmHg carried a 2.9 times increased risk for all-cause mortality than those without. Our results are in accordance with a study conducted by Inrig *et al*., which reported that an increase in SBP of more than 10 mmHg during HD was associated with decreased 2-year overall survival but was limited to patients with predialysis SBP < 120 mmHg. Although their study was conducted in a larger cohort of 1748 incident HD patients, the baseline BP measurements were averaged from only 3 consecutive HD sessions [1]. In contrast, our results were derived from the mean values of SBP recorded for 2 months. When plotting a histogram of postdialysis SBP change for each patient, the histogram derived from more times of HD sessions (*n *= 25) should more closely resemble a normal distribution. Therefore, patient grouping might be different while average postdialysis BP change was derived from different numbers of HD times. Moreover, the averaged predialysis SBP of the majority of our patients were more than 120 mmHg, which might also explain why we did not find such predictive value when categorizing our patients by 10-mmHg threshold.

Previous studies have shown that BP measured at the 44-hour interdialysis period, including ambulatory BP monitoring and self-measurement by the patient using home BP monitoring, correlates well with left ventricular hypertrophy and is a predictor of cardiovascular and all-cause mortality [14,16]. Although interdialysis ambulatory BP measurement is of greater prognostic value than HD unit BP recordings, it requires equipment and is not readily available in most HD units [19]. In addition, accurate self-measurement by the patient using home BP monitoring during the interdialysis period may be difficult for elderly and frail patients. On the other hand, peridialysis BP measurements, which are performed by dialysis unit staff shortly before and after the HD session, are much easily assessible. Furthermore, Van Buren *et al*. recently unveiled the linkage between intradialytic hypertension and interdialytic ambulatory BP burden. Their findings explain the increased morbidity and mortality seen in patients with intradialytic hypertension [23].

Although peridialysis BP recorded from 3-6 HD sessions have been reported to be poorly correlated with end-organ damage and cardiovascular outcomes [14-16], the discrepancy was suggested to result from the highly variable nature of peridialysis BP levels [19,24,25]. We believe that a high variability of peridialysis BP recordings can be offset by increasing the number of recording times. In addition, a previous study also showed that average predialysis systolic BP taken more than 1 month may be equally representative of the true BP than 24-hour ambulatory BP monitoring [17]. Therefore, we suggest that peridialysis BP averaged from 25 consecutive HD sessions is more reproducible and practical to reflect the actual postdialysis BP alterations in chronic HD patients. On the other hand, categorizing patients by a 5-mmHg interval which was averaged from a 2-month BP data might hinder its clinical applicability. Furthermore, it should be emphasized that a rise in postdialysis SBP can not be concluded from one single HD session BP recording.

There are emerging data showing that postdialysis hypertension is associated with poor outcomes, but the mechanisms of such poor outcomes remain poorly understood [1,26]. Our results revealed that postdialysis SBP rise was associated with fluid overload, as evidenced by a higher CT ratio, lower ultrafiltration volume, and lower hemoglobin level. Therefore, postdialysis SBP rise may be an implication of volume excess. In patients suffering from postdialysis hypertension, clinicians should prudently assess their volume status and intensify fluid removal as needed. Nevertheless, since both postdialysis SBP rise and high CT ratio were independent outcome predictors, there must be a mechanism other than volume excess contributing to postdialysis hypertension, such as elevated circulating endothelin and intradialytic sodium load. Therefore, drugs that inhibit the renin-angiotensin system, such as angiotensin converting enzyme (ACE) inhibitors or angiotensin II-receptor blockers (ARB) should be preferred because they cause greater regression of LVH, reduce sympathetic nerve activity, reduce pulse wave velocity, may improve endothelial function, and may reduce oxidative stress [12].

Our multivariate analysis also revealed that high UF volume had an independent association with mortality, which was consistent with previous studies showing the mortality predictive value of increased interdialytic weight gain [27,28]. However, we found patients with postdialysis SBP rise had the lowest UF volume. As mentioned above, this patient group may be volume overloaded. It seems possible that volume excess in this patient group was caused by low UF volume. However, such interpretation was limited by the lack of assessment of patient dry weight in our study. The present data suggest that both high UF volume and postdialysis SBP rise were both independent outcome predictors.

There are some limitations to our study. First, we did not include intradialytic BP in our study. However, a postdialysis BP rise may be derived from intradialytic BP elevation. Second, the sample size of our cohort is relatively small, but the BP values were averaged from 25 consecutive HD sessions during a 2-month period. Third, the positive correlation between postdialysis SBP rise and volume excess is cross-sectional rather than longitudinal. Fourth, because of the non-randomized nature of our study design, there is an excess of malignancy in the patient group with a SBP rise. Nevertheless, the independent predictive value of postdialysis SBP rise was evidenced by the multivariate analysis. Finally, the event rate was not optimal for multivariate regression analysis because of the relatively small patient number in the present study. Therefore, further large scale longitudinal studies are needed to investigate the impact of volume reduction on postdialysis SBP alterations and patient outcomes.

## Conclusions

The findings of the present study indicated that a rise in postdialysis SBP in chronic HD patients was associated with a higher risk of both long-term cardiovascular and all-cause mortality throughout the 4 years of follow-up. Since postdialysis BP rise might reflect subclinical volume excess, we suggest intensive evaluation of volume status in such patients in order to improve their survival. In addition, future studies are warranted to examine benefits of optimizing ACE inhibitor or ARB use in patients with postdialysis SBP rises.

## Competing interests

The authors declare that they have no competing interests.

## Authors' contributions

CY, WY, and YL designed research; CY performed research; CY and YL analyzed data; and CY and YL wrote the paper. All authors read and approved the final manuscript.

## Pre-publication history

The pre-publication history for this paper can be accessed here:

http://www.biomedcentral.com/1471-2369/13/12/prepub
